# One-step electrodeposition of a polypyrrole/NiO nanocomposite as a supercapacitor electrode

**DOI:** 10.1038/s41598-022-07483-y

**Published:** 2022-03-04

**Authors:** Jehan El Nady, Azza Shokry, Marwa Khalil, S. Ebrahim, A. M. Elshaer, M. Anas

**Affiliations:** 1 Electronic Materials Department, Advanced Technology and New Materials Research Institute, City of Scientific Research and Technological Applications (SRTA-City), P.O. Box 21934, New Borg El-Arab City, Alexandria Egypt; 2 Department of Materials Science, Institute of Graduate Studies and Research, Alexandria University, 163 Horrya Avenue, P.O. Box832, El-Shatby, Alexandria Egypt; 3 Nanotechnology and Composite Materials Department, Advanced Technology and New Materials Research Institute, City of Scientific Research and Technological Applications (SRTA-City), P.O. Box 21934, New Borg El Arab City, Alexandria Egypt; 4 Department of Computer Engineering, Higher Institute of Engineering and Technology, P.O. Box 22751, El-Boheira, Egypt; 5 Physics Department, Faculty of Science, Alexandria University, Moharram Bek, Alexandria, 21511 Egypt

**Keywords:** Energy science and technology, Materials science, Nanoscience and technology

## Abstract

An electrochemical deposition technique was used to fabricate polypyrrole (Ppy)/NiO nanocomposite electrodes for supercapacitors. The nanocomposite electrodes were characterized and investigated by Fourier transform infrared spectroscopy (FTIR), X-ray Diffraction (XRD), scanning electron microscopy (SEM), cyclic voltammetry (CV), galvanostatic charge–discharge (GCD) and electrochemical impedance spectroscopy (EIS). The performance of supercapacitor electrodes of Ppy/NiO nanocomposite was enhanced compared with pristine Ppy electrode. It was found that the Ppy/NiO electrode electrodeposited at 4 A/cm^−2^ demonstrated the highest specific capacitance of 679 Fg^−1^ at 1 Ag^−1^ with an energy density of 94.4 Wh kg^−1^ and power density of 500.74 W kg^−1^. Capacitance retention of 83.9% of its initial capacitance after 1000 cycles at 1 Ag^−1^ was obtained. The high electrochemical performance of Ppy/NiO was due to the synergistic effect of NiO and Ppy, where a rich pores network-like structure made the electrolyte ions more easily accessible for Faradic reactions. This work provided a simple approach for preparing organic–inorganic composite materials as high-performance electrode materials for electrochemical supercapacitors.

## Introduction

Supercapacitors, as storage devices, play a main role in bridging the gap between conventional capacitors and batteries^1^. Conducting polymers can be used as electrodes to compensate for the low energy density of carbon structures because of their highly reversible oxidation–reduction, pseudocapacitance and high electrical conductivity^2,3^. The pseudosupercapacitor is of interest due to its high power and energy density. Ppy, polyvinylcarbazole, polythiophene, polyazulene and polyaniline have been applied as electrodes for pseudocapacitors. Ppy has many advantages where its pyrrole monomer is easily oxidized and soluble in water, and Ppy has a reversible electrochemical doping/dedoping process^4–6^.

The fabrication of Ppy electrodes by electropolymerization of pyrrole is an interesting technique that allows pyrrole and certain dopants to be oxidized at the electrode surface by applying an anodic potential or a current or potentiodynamic window (cyclic voltammetry) to form a polymer film^7^. The dopant is inserted during Ppy formation to confirm the electrical neutrality of the produced film. Different works have focused on the modification of Ppy, optimization of film deposition conditions, and development of dopants^8,9^. High electrical conductivity and thermal stability of Ppy films were obtained using aromatic anionic dopants. The electrical and electrochemical properties were modified and controlled using certain functionalized dopants, such as p-toluene sulfonate, anthraquinone-2-sulfonate and benzene sulfonate^10^. However, Ppy undergoes deformation during cyclic voltammetry and charge–discharge, which leads to a reduction in cyclic stability. To enhance the stability of Ppy, composites with hierarchical structured nanomaterials reduce the volume change of Ppy during charge–discharge^11^. In addition, different transition metal oxides can be considered as electrodes pseudocapacitor behavior^12,13^. Compared with the electrode materials based on carbon derivatives or conducting polymers, transition metal oxides (TMOs) possess the advantage of higher capacitances in practice^14–16^. NiO is a promising pseudocapacitive electrode due to its high capacity and stability. On the other hand, the performance of NiO electrodes is poor due to their low conductivity^17,18^. In this study, a new nanocomposite of Ppy/NiO electrodes were fabricated via one–step facile electrochemical deposition method at different currents onto the surface of graphite sheet. The electrochemical behavior of the as-prepared electrodes was investigated by cyclic voltammetry (CV), galvanostatic charge–discharge (GCD) and electrochemical impedance spectroscopy (EIS).

## Method

### Materials

Pyrrole monomer (Py, 98%) was obtained from Sigma-Aldrich, Germany. Nickel sulfate (NiSO_4_) and nickel chloride (NiCl_2_) were purchased from LOBAL Chemie, Mumbai, India. Boric acid (H_3_BO_3_) and lithium perchlorate (LiClO_4_) were received from local chemical companies. A graphite sheet (GS) and platinum rod (Pt) was purchased from Shanghai Phoenix Alloy Co., China. Hydrochloric acid (34%) and ethanol (99.8%) were obtained from Alfa Aesar and J. T. Baker, respectively. Dodecylbenzene sulfonic acid (DBSA, C_18_H_30_O_3_S) was received from El-Gomhoria Chemical Company, Egypt. All chemicals were used without further purification.

### Preparation of Ppy_1%_-DBSA_2%_/NiO_97%_-GS supercapacitor electrodes

Ppy_1%_-DBSA_2%_/NiO_97%_-GS supercapacitor electrodes were synthesized by electrochemical deposition using nickel sulfate, nickel chloride, and boric acid as precursors for NiO formation and DBSA and Py as sources of doped Ppy. Typically, 15.5 g of NiSO_4_, 2.5 g of NiCl_2_, and 2 g of H_3_BO_3_ were dissolved in 50 mL deionized water with stirring for 1 h to form a foam light green solution. Then, 1 mL Py and 2 mL DBSA were added to the light green solution and stirred for 1 h at room temperature until a heavily green solution appeared. The electrochemical deposition supercapacitor (SC) electrodes were synthesized using an electrochemical OrigaFlex-OGF05 (Origalys, France) workstation with different currents using a three-electrode cell configuration at room temperature. The three electrode cell contains a 4.0 × 1.5 cm^2^ Pt sheet as a counter electrode and Ag/AgCl as a reference electrode. The GS substate was cut into rectangular shapes with dimensions of 4.5 × 1 × 0.3 cm^3^ as a current collector. The Ppy_1%_-DBSA_2%_/NiO_97%_-GS electrode was synthesized by depositing a thin layer using chronopotentiometry at current densities of 2, 4, 6, 8, and 10 mA cm^−2^ for 10 min. The SC electrodes prepared at various currents of 2, 4, 6, 8, and 10 mA cm^−2^ were coded as Ppy_1%_-DBSA_2%_/NiO_97%_-GS@2, Ppy_1%_-DBSA_2%_/NiO_97%_-GS@4, Ppy_1%_-DBSA_2%_/NiO_97%_-GS@6, Ppy_1%_-DBSA_2%_/NiO_97%_-GS@8, and Ppy_1%_-DBSA_2%_/NiO_97%_-GS@10, respectively. The fabricated SC electrodes were rinsed with deionized water and dried at 60 °C.

### Characterization

The composition of the prepared Ppy_1%_-DBSA_2%_/NiO_97%_-GS supercapacitor electrode was studied using Fourier transform infrared (FTIR) (Bruker Corporation, Ettlingen, Germany). The crystalline structure of Ppy/NiO nanocomposite and PPy were studied by using X-ray diffraction was performed using (X-ray 7000 Shimadzu-Japan) at room temperature in the range of 2 h from 10° to 100°. The X-ray source Cu target generated at 30 kV and 30 mA with a scan speed of 4° min^−1^. The morphological properties of the prepared nanocomposite were investigated using scanning electron microscopy (SEM), JEOL (JSM 6360 LA, Japan) instruments. Galvanostatic electrochemical charge/discharge (GCD), cyclic voltammetry (CV) and electrochemical impedance spectroscopy (EIS) measurements were performed in a three electrode cell at room temperature using a computer-controlled potentiostat (Metrohm Autolab 87070, Germany). Pt and Ag/AgCl electrodes were immersed in acetonitrile of 0.1 M LiClO_4_ electrolyte solution in the three electrode cell to characterize the fabricated Ppy_1%_-DBSA_2%_/NiO_97%_-GS SC electrodes. CV and GC/D were carried out for the fabricated SC electrodes in the potential window ranging from 0 to 1 V at sweep rates of 5, 15, 35, 50, 75, and 100 mV s^−1^ and current densities from 1 to 3 Ag^−1^. EIS was implemented in the frequency range from 100 kHz to 0.01 Hz at 5 mV. Cyclic stability test was conducted at a current density of 1 Ag^−1^ for 1000 cycles. Different performance parameters of the specific capacitance (Cs), energy density (E) and power density (P) of the prepared SC electrodes were calculated by the following equations^19,20^:

1 $$Cs=\frac{{\int }_{Vi}^{Vf}I\left(V\right)dV}{2\mathrm{ s m }\Delta\mathrm{V}}$$

2 $$\mathrm{Cs}=\frac{\mathrm{I}\times \mathrm{t}}{\mathrm{m}\times \mathrm{\Delta V}}$$

3 $$E=\frac{Cs\times \Delta {V}^{2}}{2\times 3600}$$

4 $$P=\frac{E\times 3600}{\Delta t}$$

where I, t, ΔV, s, and m are the discharge current (A), discharge time (s), discharge potential window (V), scan rate (V s^−1^) and mass of the active material (g), respectively.

## Results and discussion

### Electropolymerization of Ppy_1%_-DBSA_2%_/NiO_97%_-GS

**Figure 1:**
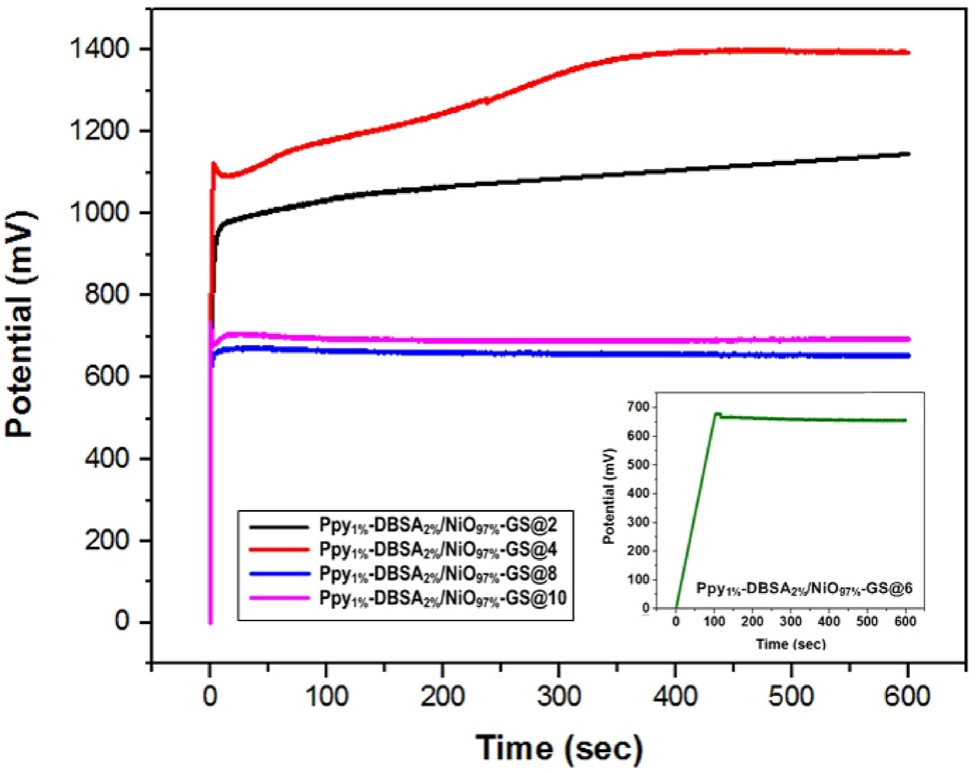
Potential versus time curves of the chronopotentiometry processes of Ppy_1%_-DBSA_2%_/NiO_97%_-GS at different current densities from 2 to 10 mA cm^−2^.

Figure 1 shows the potential-time curves of the chronopotentiometry processes of the synthesis of Ppy_1%_-DBSA_2%_/NiO_97%_-GS with different current densities from 2 to 10 mA cm^−2^ for 600 s. The electropolymerization process usually consists of three stages, as reported in the literature^21–23^. At the beginning of electropolymerization deposition, the voltage suddenly increases after application of different current densities due to the cathodic overpotential between Pt as a counter and GS as a working electrode. The maximum potentials recorded in the voltage–time curves of the prepared Ppy_1%_-DBSA_2%_/NiO_97%_-GS film at 2, 4, 6, 8, and 10 mA cm^−2^ are 1045, 1350, 678, 680, and 717 mV, respectively. For 2 and 4 mA cm^−2^ the voltage increases to the supersaturation region with time due to the increases in the number of charge carriers and the critical grain size or the formation of oligomers of the Ppy_1%_-DBSA_2%_/NiO_97%_-GS deposited on the GS electrode. This can be explained based on the first few seconds of the applied current is sufficient to form the radical cations on the pyrrole rings and start the propagation step in the stage of growth. The potential needs for the formation of radicals from the dimer or trimer is lower than that required to form the pyrrole radicals. Consequently, the potential is lower again after few seconds and attain to the plateau regions. Moreover, the temporary decays appear as a small valley in the voltage curves at 6, 8, and 10 mA cm^−2^, which correspond to the diffusion limitation of the oxidation process on the Py monomer, and then small peaks appear after the valley is revealed to the nucleation end growth of the Ppy^21,22^. It is noted that at 2 mA cm^−2^, the saturation potential is about 1000 mV and with increasing the current density to 4 mA cm^−1^ the saturation potential is raised to about 1200 mV. This indicates the formation of the DBSA doped Ppy film, which is more conductive than the GS substrate However, at 8 and 10 mA cm^−2^ the plateau region is declined to 700 and 750 mV, respectively and this is attributed to the degradation of the polymeric films at high current density.

### Structural analysis of Ppy_1%_-DBSA_2%_/NiO_97%_-GS film

**Figure 2:**
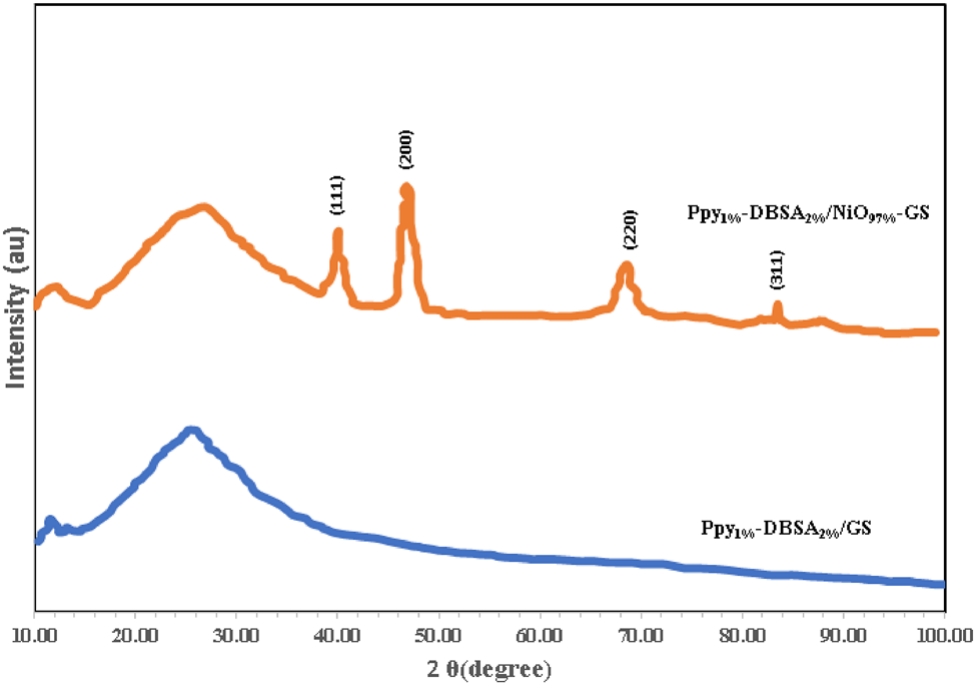
XRD patterns of pure Ppy_1%_-DBSA_2%_/GS and Ppy_1%_-DBSA_2%_/NiO_97%_-GS.

The XRD patterns of pristine Ppy_1%_-DBSA_2%_/GS and Ppy_1%_-DBSA_2%_/NiO_97%_-GS electrodes are shown in Fig. 2. The XRD pattern of pure Ppy_1%_-DBSA_2%_/GS displays a broad peak at 2θ = 25.20°^24^. The XRD pattern of the Ppy_1%_-DBSA_2%_/NiO_97%_-GS shows all the characteristic sharp peaks of a cubic phase of NiO (JCPDS: 47-1049)^25–28^. The characteristic peaks at 2θ = 39.50°, 46.80°, 68.60° and 83.50° can be indexed to (111), (200), (220), (311) and (222) diffraction planes of NiO, respectively^29,30^. The XRD pattern represents the formation of NiO/PPy composite with good crystal phase.

**Figure 3:**
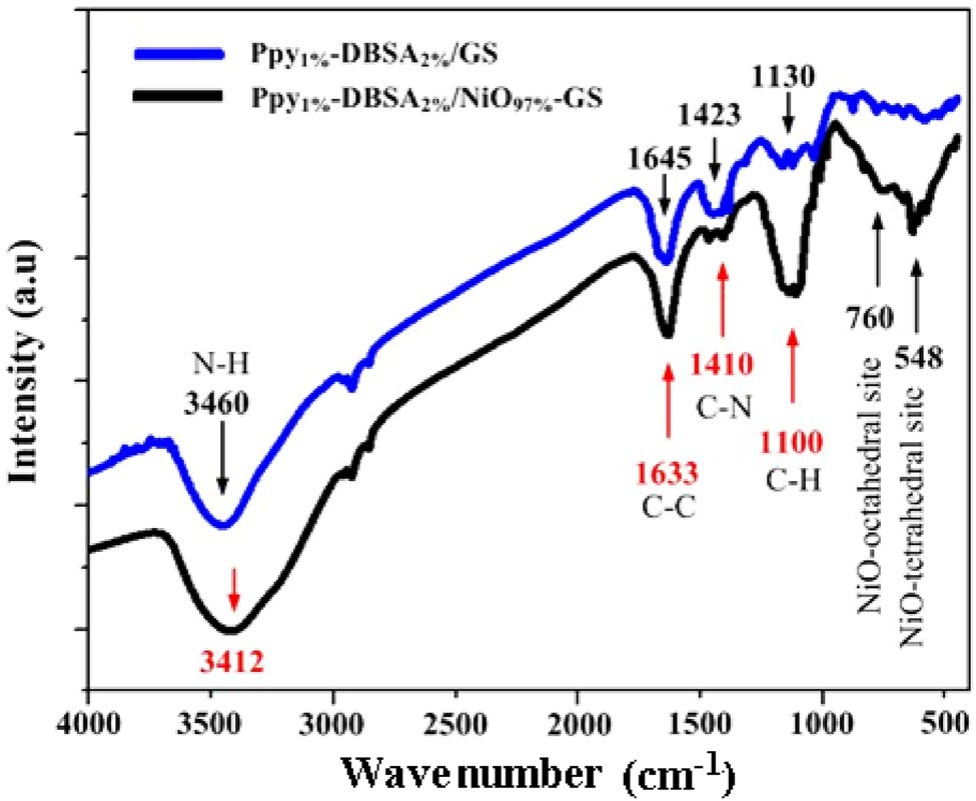
FTIR spectra of pure Ppy_1%_-DBSA_2%_/GS and Ppy_1%_-DBSA_2%_/NiO_97%_-GS.

Figure 3 presents the FTIR spectra of pure Ppy_1%_-DBSA_2%_/GS and Ppy_1%_-DBSA_2%_/NiO_97%_-GS in the range of 400–4000 cm^–1^. A broad band at approximately 3460 cm^–1^ is attributed to N–H stretching associated with the bound pyrrole ring^24^. The peaks at 1645, 1423, and 1130 cm^–1^ are assigned to the C–C and C–N stretching vibrations and the C–H in-plane vibrational bands of the polypyrrole ring, respectively^25^. These results indicate that Ppy has been formed. In the Ppy_1%_-DBSA_2%_/NiO_97%_-GS sample, the observed absorption bands at 760 and 548 cm^−1^ are corresponded to the torsional and stretching vibration modes of the NiO bond at the octahedral and tetrahedral sites, respectively^26,27^. Most of the stretching vibrations in the Ppy_1%_-DBSA_2%_/NiO_97%_-GS sample are the same as that of PPy, with only a slight shift of IR absorption to lower frequencies in Ppy_1%_-DBSA_2%_/NiO_97%_-GS to 3414, 1633, 1410, and 1100 cm^−1^, respectively. This suggests that an interaction between the polymer and NiO occurs. This change is due to loss in conjugation and molecular order after modification of Ppy with NiO. This result indicates a strong interaction between Ppy and NiO nanoparticles^28,29^.

### Surface morphology analysis

**Figure 4:**
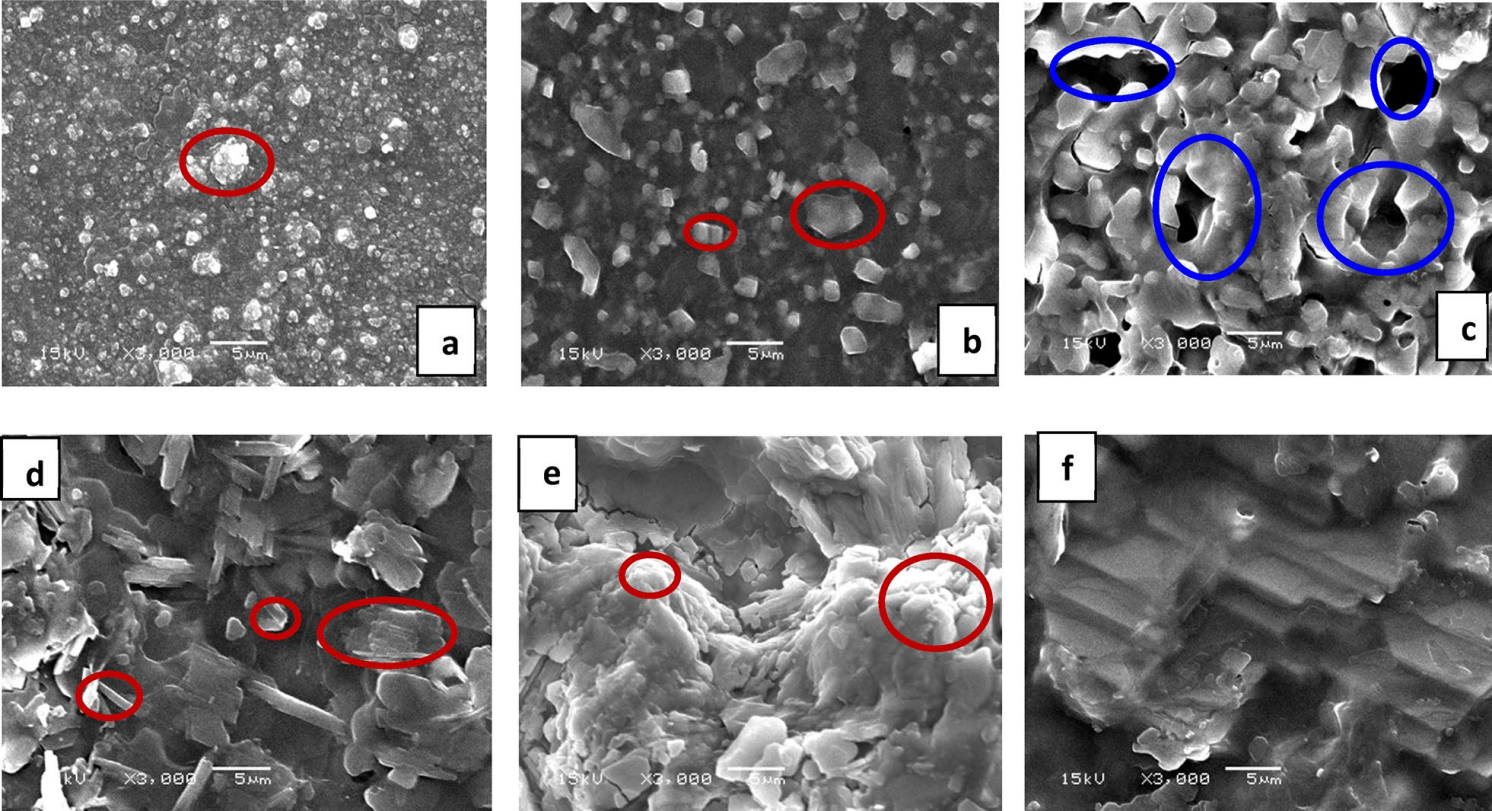
SEM images of (**a**) pure Ppy_1%_-DBSA_2%_/GS, (**b**) Ppy_1%_-DBSA_2%_/NiO_97%_-GS@2, (**c**) Ppy_1%_-DBSA_2%_/NiO_97%_-GS@4, (**d**) Ppy_1%_-DBSA_2%_/NiO_97%_-GS@6, (**e**) Ppy_1%_-DBSA_2%_/NiO_97%_-GS@8, and (**f**) Ppy_1%_-DBSA_2%_/NiO_97%_-GS@10.

SEM micrographs of the electrochemically prepared pure Ppy_1%_-DBSA_2%_/GS film are illustrated in Fig. 4a. The electronic properties of polypyrrole films are linked to their morphology; the smoother and denser surface leads to a conductive film, and the more pores and ordered film facilitates charge transfer through the film. Ppy films show a cauliflower-like nodular surface morphology and microspherical grains of approximately less than 1 μm diameter. The effect of current densities and NiO on the microstructure of Ppy is indicated in Fig. 4b–e. There are a number of faceted grains elongated and rectangular blocks with different dimensions observed. These blocks result from the formation of NiO, as shown in Fig. 4b. Increasing the current density to 4 mA cm^−2^ for prepared in situ Ppy and NiO, as presented in Fig. 4c, drives to fusion of the cauliflower and rectangular structures and formation of pores, voids and compatible phases. The rich pores like-structure facilitates the diffusion and transfer of ions from the electrolyte to the electrode film and vice versa. On the other hand, for Ppy_1%_-DBSA_2%_/NiO_97%_-GS@6 displayed in Fig. 4d, the increasing of the current to 6 mA results in a higher thickness of the nanocomposite and induce the phase separation between PPy and NiO. For this composite, some of the rectangular blocks are converted to long rods. For PPy_1%_-DBSA_2%_/NiO_97%_-GS@8, the dominant phase is a cauliflower-like structure of PPy. Finally, at 10 mA for electrodeposition of the nanocomposite electrode of PPy/NiO, graded stairs layers are obtained, as illustrated in Fig. 4f.

### Electrochemical Properties of Ppy/NiO electrodes

**Figure 5:**
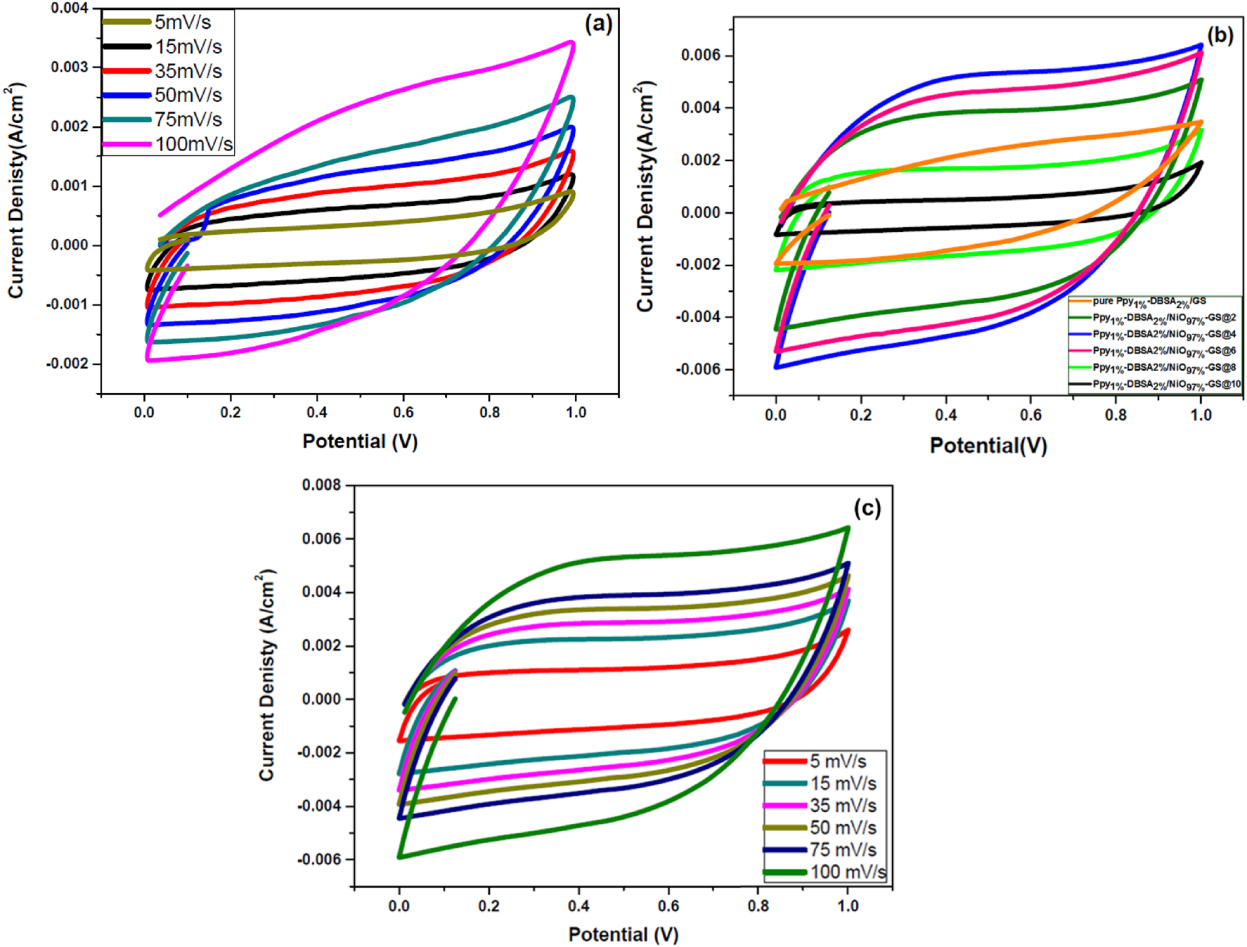
CV curves of (**a**) pure Ppy_1%_-DBSA_2%_/GS at different scan rates, (**b**) Ppy_1%_-DBSA_2%_/GS and Ppy_1%_-DBSA_2%_/NiO_97%_-GS nanocomposites at 100 mV/s, and (**c**) Ppy_1%_-DBSA_2%_/NiO_97%_-GS@4 at different scan rates.

The CV measurements for pure Ppy_1%_-DBSA_2%_/GS and Ppy_1%_-DBSA_2%_/NiO_97%_-GS electroplated electroplated with current densities in 0.1 M LiClO_4_ electrolyte solution and at different scan rates are displayed in Fig. 5. CV curves of pristine Ppy_1%_-DBSA_2%_/GS (shown in Fig. 5a) at different scan rates ranging from 5 to 100 mV s^−1^ exhibit nearly rectangular shapes symmetrical across the zero current axis, and they do not appear to be oxidation–reduction peaks^31,32^, showing the typical characteristic of electrical double layer capacitance^33,34^. It is observed that the initial or start point of CV cycle toward anodic direction is not the same the end or final point during reversed in the cathodic direction. This can be explained based on the irreversibility of oxidation reduction reaction or due to effect of high scan rate. The current density of Ppy_1%_-DBSA_2%_/NiO_97%_-GS supercapacitor electrode is directly proportional to scan rate. After the incorporation of NiO_97%_ into Ppy_1%_-DBSA_2%_/GS at different electroplating current densities, as shown in Fig. 5b, the integrated area of the CV curves of the electroplated electrode significantly increases due to the high pseudocapacitance originating from NiO, high specific surface area, and abundant redox active sites generated by the porous NiO network^33,35^. The CV curves have a large enclosed area and good symmetrical rectangular shape, showing that the capacitive behavior of the electrode could be greatly improved by optimizing the electrodeposition current of the Ppy_1%_-DBSA_2%_/NiO_97%_-GS composite. The Ppy_1%_-DBSA_2%_/NiO_97%_-GS@4 supercapacitor electrode produces the maximum enclosed area. The CV curves of the Ppy_1%_-DBSA_2%_/NiO_97%_-GS@4 supercapacitor electrode at various scan rates from 5 to 100 mV s^−1^ are displayed in Fig. 5c. A quasi-rectangular shape with no distortion appears in the CV curves. In addition, the current densities linearly increase with increasing scan rates, which may be attributed to the confirmation of the formation of efficient electrical double layers and fast charge propagation within the electrodes^36^.

**Figure 6:**
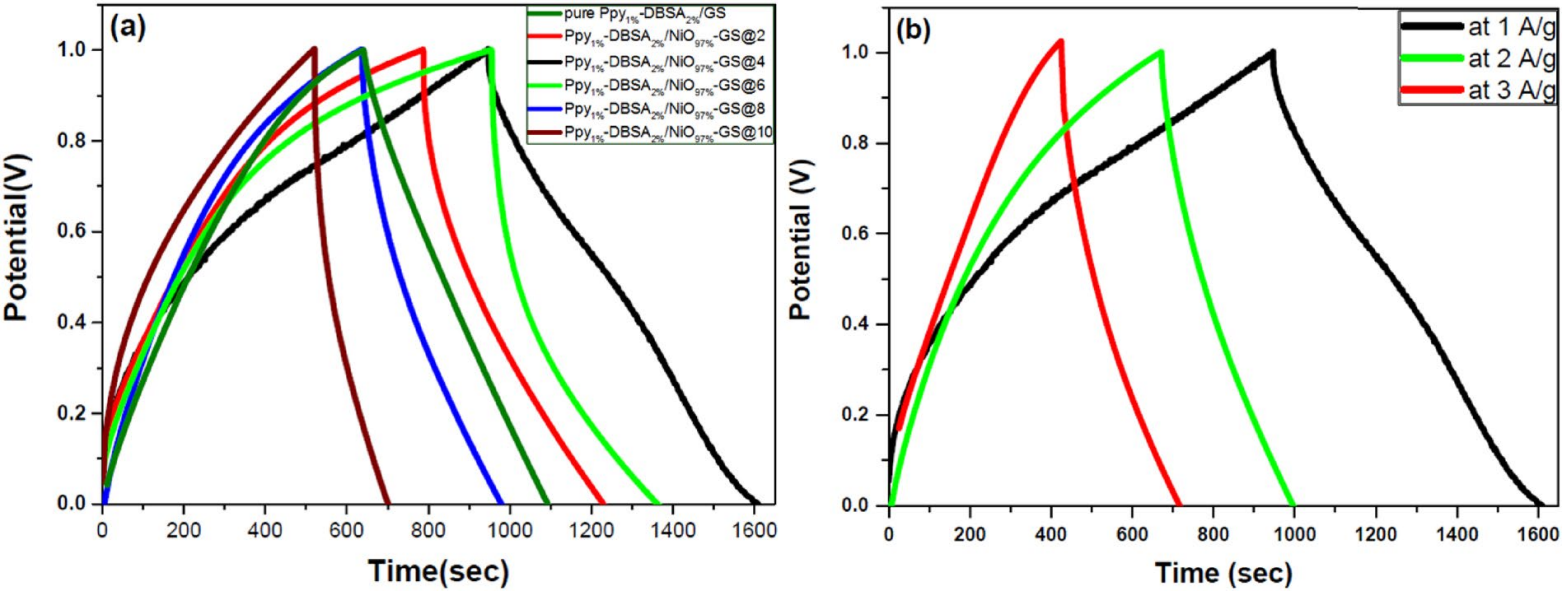
GCD curves of (**a**) pure Ppy_1%_-DBSA_2%_/GS and Ppy_1%_-DBSA_2%_/NiO_97%_-GS nanocomposite electrodes at 1 A g^−1^ and (**b**) Ppy_1%_-DBSA_2%_/NiO_97%_-GS@4 at different currents.

The GCD curves of pure Ppy_1%_-DBSA_2%_/GS and Ppy_1%_-DBSA_2%_/NiO_97%_-GS supercapacitor electrodes prepared with different current densities and measured at 1 A/g are presented in Fig. 6a. It is noted that symmetric triangular curves for fabricated supercapacitor electrodes and low charge-transfer resistances during charging and discharging even at high current densities with very little ohmic drop are obtained, indicating a high rate of their performance^37^. It is also found that the discharge time of the Ppy_1%_-DBSA_2%_/NiO_97%_-GS@4 supercapacitor electrode is the highest among the other electrodes prepared with different current densities. Consequently, the GCD curves for Ppy_1%_-DBSA_2%_/NiO_97%_-GS@4 at 1.0, 2.0 and 3.0 A g^−1^ are shown in Fig. 6b, through which good linear potential-time profiles are achieved, demonstrating the good capacitance performance of this electrode. The specific capacitances are found to be 679, 333.5 and 292.7 F g^−1^ at 1, 2 and 3 A g^−1^, respectively, showing the rate capability of the synthesized sample Ppy_1%_-DBSA_2%_/NiO_97%_-GS@4^38^. Using Eqs. (3) and (4), the energy density of 94.4 Wh kg^−1^ and power density of 500.74 W kg^−1^ are obtained for the highest capacitance sample, Ppy1%-DBSA2%/NiO97%-GS@4 and this considers large values compared with other Ppy/NiO supercapacitors electrodes in the literatures^16,24,25,40,41^. The denser and more compact structure may have prevented cations from migrating into the electrode material^39^. For this reason, the specific capacitance of the composites first increases and then decreases with increasing Ppy/NiO film thickness^40^. Ppy_1%_-DBSA_2%_/NiO_97%_-GS@4 (679 Fg^−1^) shows the highest performance compared with the other composites due to its high porosity, as observed in the SEM image (Fig. 4c). This provides paths to diffuse electrolyte ions into the hybrid arrays and enhances the Faradaic reactions^41^. Ppy_1%_-DBSA_2%_/NiO_97%_-GS@10 (170 F g^−1^) exhibits the smallest value of the specific capacitance, as shown in Fig. 6a, which is in good agreement with the CV results. The specific capacitance of Ppy_1%_-DBSA_2%_/NiO_97%_-GS@4 is larger than that of pure PPy (456 F g^−1^), and this is attributed to the synergistic effect of NiO and Ppy. The embedment of NiO as a molecular level dispersion in the Ppy matrix can reduce electron shuttling along the conjugated chains by interlinking the Ppy chains, leading to the enhancement of the overall conductivity of NiO/PPy^39^.

**Figure 7:**
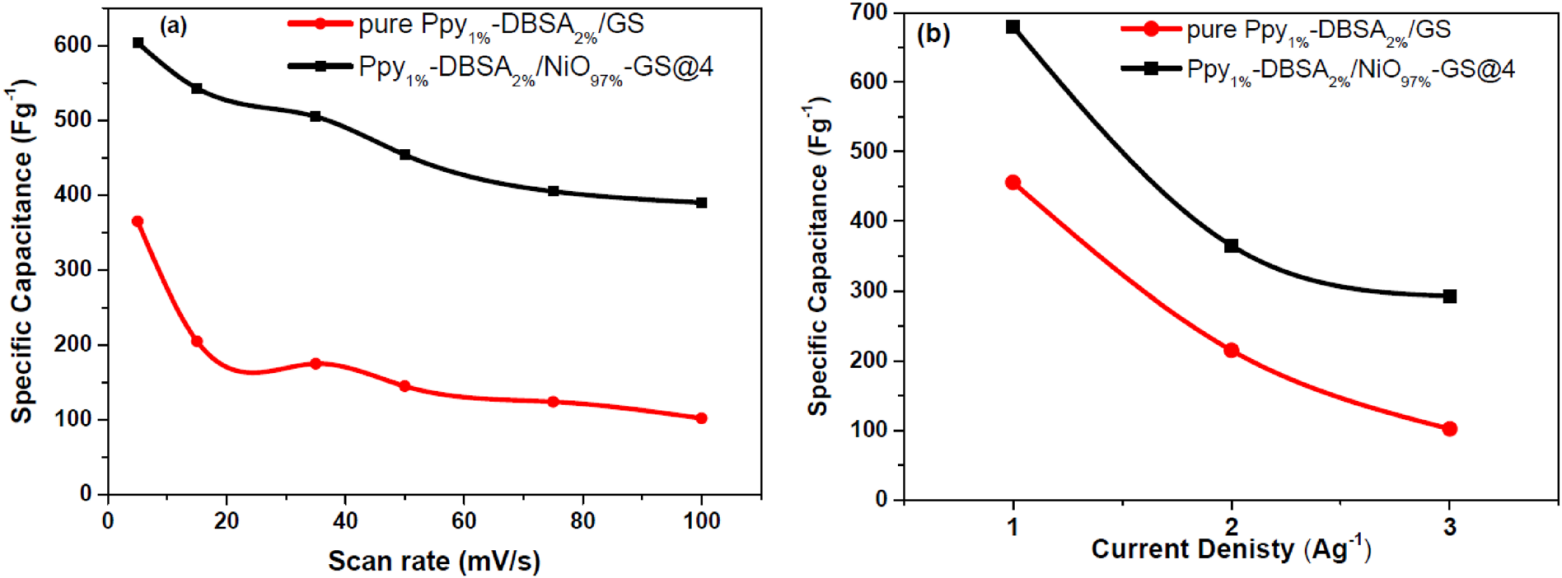
Specific capacitance at different scan rates (**a**) and specific capacitance at different current densities (**b**) for pure Ppy_1%_-DBSA_2%_/GS and Ppy_1%_-DBSA_2%_/NiO_97%_-GS@4.

The specific capacitance (Csp) was calculated from the CV curves by integrating the area under the CV curve using Eq. (1). As shown in Fig. 7a, Ppy_1%_-DBSA_2%_/NiO_97%_-GS@4 has larger Csp values than Ppy_1%_-DBSA_2%_/GS at different scan rates from 5 to 100 mV s^−1^. The Csp value of Ppy_1%_-DBSA_2%_/NiO_97%_-GS@4 at 5 mV s^−1^ is calculated to be 605 F g^−1^ and is higher than the value of 364 F g^−1^ for Ppy_1%_-DBSA_2%_/GS at the same scan rate. The dependence of Csp on scan rate exhibits a decay of 35% Csp of Ppy_1%_-DBSA_2%_/NiO_97%_GS@4 with increasing scan rate from 5 to 100 mV s^−1^. The area under the CV curves increases. It is noted that the shape of CVs at different scan rates is the same indicating the excellent rate capability and reversibility of the SC electrodes. At low scan rate, the electrolyte ions diffuse and migrate into active Ppy and high specific capacitances are produced. On the other hand, the lower specific capacitances of SC electrode at high scan rate are attributed to inaccessibility of electrolyte ions to some active surface sites. Even at a scan rate as high as 100 mV s^−1^, the Ppy_1%_-DBSA_2%_/NiO_97%_-GS@4 electrode still achieves a Csp value as large as 359 F g^−1^. However, for Ppy_1%_-DBSA_2%_/GS, Csp shows a severe decay of 73% with increasing scan rate from 5 to 100 mV s^−1^, and Csp is only 99.3 F g^−1^ at 100 mV s^−1^.

Figure 7b illustrates the Csp values at various current densities from 1 to 3 A g^−1^. Csp values can be derived from GCD curves by using Eq. (2). The Ppy_1%-_DBSA_2%_/NiO_97%_-GS@4 sample shows much larger Csp values at all current densities than Ppy_1%_-DBSA_2%_/GS with current densities increasing from 1 to 3 A g^−1^, which is in agreement with the results obtained from CV tests. The Csp of Ppy_1%-_DBSA_2%_/NiO_97%_-GS@4 at 1 A g^−1^ is calculated to be 679 F g^−1^, which is much larger than the value of 456 F g^−1^ for Ppy_1%_-DBSA_2%_/GS. Depending on these results calculated above, the capacitance utilization of Ppy_1%-_DBSA_2%_/NiO_97%_-GS@4 is higher than that of Ppy_1%_-DBSA_2%_/GS, indicating that a homogeneous distribution of PPy and NiO particles is beneficial for the transport of ions in full-gapped nanoparticle systems and for the increase of the PPy/electrolyte interfacial area. The decline of the specific capacitance at elevating current density is due to the inaccessibility of electroactive sites by the electrolyte ions.

**Figure 8:**
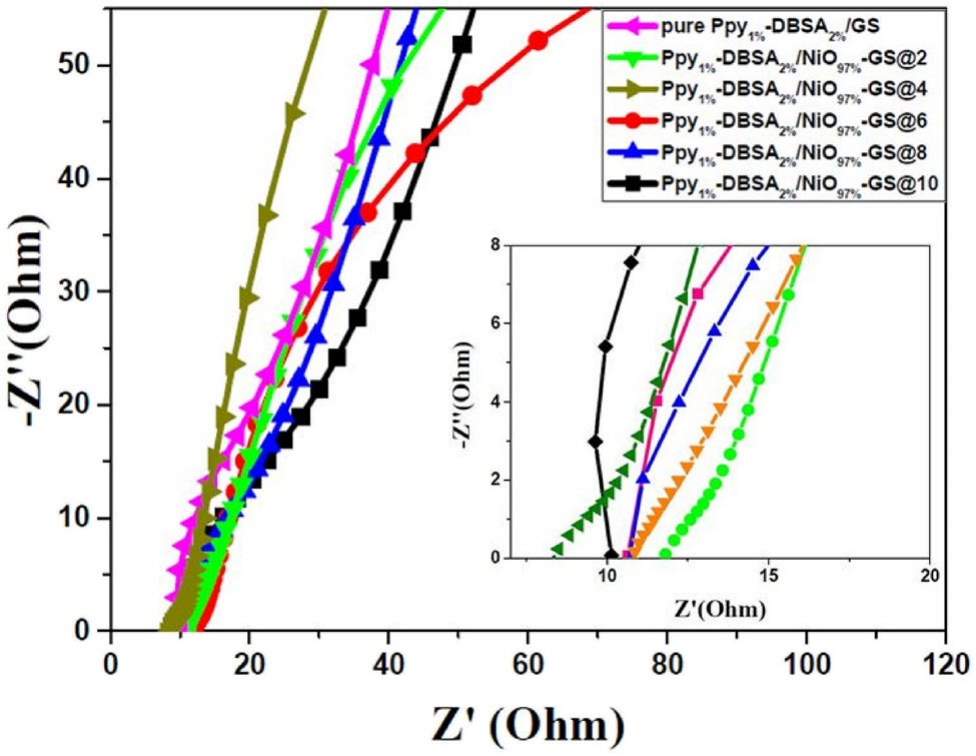
Nyquist plots (real impedance vs. imaginary impedance) of PPy/NiO electrodes with different currents in the frequency range of 0.01 Hz to100 kHz.

Figure 8 shows the Nyquist plots for the Ppy/NiO nanocomposite electrodes synthesized electrochemically with different currents at the frequency range from 0.01 to100 kHz with amplitude of 5 mV. The long tails in the low-frequency region or the diffusion region are nearly vertical to the real axis. The intercept of the high-frequency curve in the real part reflects the equivalent series resistance (Rs) between the electrodes and electrolyte and equal to the summation of the Ohmic resistance of the electrolyte, the contact resistance, and the internal resistance of the material. From the inset of Fig. 8, the Ppy1%-DBSA2%/NiO97%-GS@4 electrode possesses the smallest Rs (8.1 Ω) compared to pure Ppy1%-DBSA2%/GS (10 Ω), and Ppy1%-DBSA2%/NiO97%-GS@6 exhibits the largest Rs (11.8 Ω). However, no distinct semicircles are observed in the plots of all electrochemically prepared samples, indicating a small charge transfer resistance between the electrode and electrolyte and consequently the low effect of capacitive double layer. This resulted from the Ppy effect as a conducting polymer which proposed to have a redox behavior. Ppy1%-DBSA2%/NiO97%-GS@4 and Ppy1%-DBSA2%/NiO97%-GS@6 have more vertical slopes demonstrating that they possess low diffusion resistances and contact resistances between Ppy/NiO and the GS substrate^42,43^. These results are consistent and match well with the results obtained with SEM, CV, and GCD.

**Figure 9:**
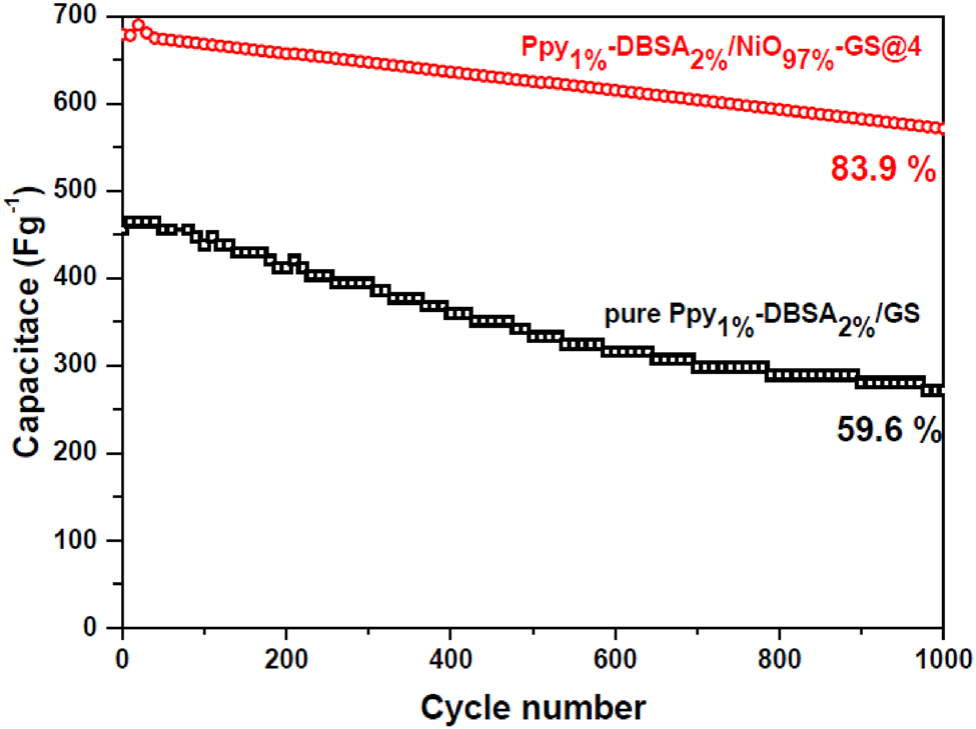
Cycling stability measurement of the pure Ppy_1%_-DBSA_2%_/GS and Ppy_1%_-DBSA_2%_/NiO_97%_-GS@4 electrodes at 1 A g^−1^ for 1000 cycles.

The cycle stability as an important parameter for supercapacitors for Ppy_1%_-DBSA_2%_/NiO_97%_-GS@4 and pure Ppy_1%_-DBSA_2%_/GS electrodes can be valued by their consecutive GCD at 1 Ag^−1^ for 1000 cycles. As shown in Fig. 9, the specific capacitance retentions are 83.9% and 59.6% of the initial value after 1000 cycles for Ppy1%-DBSA2%/NiO97%-GS@4 and pure Ppy1%-DBSA2%/GS, respectively. The Cs reduction resulted from a degradation of the PPy chains due to the excessive swelling and shrinking of the PPy polymer during the charge/discharge process. The clearly excellent long-term cycling stability of the Ppy1%-DBSA2%/NiO97%-GS@4 composite may be attributed to the porous network gapped structure and good conductivity, which were favorable for charge transportation and electrolyte diffusion^39^. The presence of NiO nanoparticles not only enhances the capacitance value but also improves the cycling stability^44^.

## Conclusion

The electrochemical results were improved for the chronopotentiometry deposited Ppy/NiO electrode at different currents onto the graphite sheet compared with the pristine Ppy electrode. It was found that the Ppy_1%_-DBSA_2%_/NiO_97%_-GS@4 electrode demonstrated a high specific capacitance of 679 Fg^−1^ at a current density of 1 Ag^−1^ and capacitance retention of 83.9% of its initial capacitance after 1000 cycles at 1 Ag^−1^. The high electrochemical performance of Ppy_1%_-DBSA_2%_/NiO_97%_-GS@4 was due to the synergistic effect of NiO and Ppy, where a uniform porous network-like structure made the electrolyte ions more easily accessible for Faradic reactions.
